# Expression of p57 Immunostain in Complete and Partial Hydatidiform Moles

**DOI:** 10.7759/cureus.102487

**Published:** 2026-01-28

**Authors:** Ummey Salma Shabnam, AKM Nurul Kabir, Tasmia Islam, Syeeda Shiraj-Um-Mahmuda, Papiya Rahman, Rezwana Karim, Mohammad Mosiur Rahman, Raisa Badhan

**Affiliations:** 1 Pathology, National Institute of Cancer Research and Hospital, Dhaka, BGD; 2 Pathology, Bangladesh Medical University, Dhaka, BGD; 3 Pathology, Directorate General of Health Services, Dhaka, BGD; 4 Pathology, US-Bangla Medical College and Hospital, Dhaka, BGD; 5 Microbiology, National Institute of Burn and Plastic Surgery, Dhaka, BGD

**Keywords:** complete mole, hydatidiform mole, immunohistochemistry, p57, partial mole

## Abstract

Introduction: Hydatidiform mole (HM) is a gestational trophoblastic disease characterized by abnormal proliferation of trophoblastic tissue. Accurate subclassification into complete hydatidiform mole (CHM) and partial hydatidiform mole (PHM) is essential, as CHM carries a higher risk of persistent trophoblastic disease and choriocarcinoma. Histopathology alone may be inconclusive due to overlapping features and interobserver variability. p57 immunohistochemistry has emerged as a valuable ancillary tool in differentiating CHM from PHM. This study aimed to evaluate p57 expression across histopathologically diagnosed cases of HM and determine its usefulness as a diagnostic marker.

Materials and methods: A cross-sectional observational study was conducted on 57 cases diagnosed as complete, partial, or indeterminate HM from the Bangladesh Medical University (BMU) and private laboratories in Dhaka. All cases were re-evaluated based on defined histopathological criteria. p57 immunohistochemistry was performed in the Department of Pathology, BMU, and staining results were compared across diagnostic groups. Statistical analysis was performed using SPSS Statistics version 22.0 for Windows (IBM Corp., Armonk, NY, USA). A p value <0.05 was considered significant.

Results: Of 57 cases, 36 were histologically diagnosed as CHM, 13 as PHM, and eight as indeterminate. Among CHM, 32 (88.9%) showed negative p57 expression, while four (11.1%) were positive. In PHM cases, nine (69.2%) showed positive expression, and four (30.8%) were negative. Among indeterminate cases, six showed negative expression, and two were positive. p57 expression demonstrated a statistically significant association with final diagnosis, leading to reclassification of cases to 42 CHM and 15 PHM.

Conclusion: p57 immunostaining significantly enhances diagnostic accuracy and reduces ambiguity in differentiating complete from partial HM. Incorporation of p57 evaluation alongside histopathology is recommended for reliable subclassification of HM.

## Introduction

Hydatidiform moles (HM) are abnormal gestational events characterized by hydropic swelling of chorionic villi and excessive trophoblastic proliferation. They fall under the spectrum of gestational trophoblastic diseases (GTD), which include premalignant entities such as HM and malignant lesions like invasive mole and choriocarcinoma [1]. Although complete HM (CHM) and partial HM (PHM) share overlapping clinical and morphological features, distinction between the two is critical because CHM carries a substantially higher risk of persistent trophoblastic disease requiring chemotherapy, whereas PHM is associated with a much lower malignant potential [2,3].

Despite the presence of certain characteristic microscopic features, such as diffuse trophoblastic proliferation, marked cytologic atypia, and cistern formation in CHM, and two populations of villi with scalloped outlines and focal trophoblastic hyperplasia in PHM, significant morphologic overlap often leads to diagnostic discrepancies. Even among experienced pathologists, interobserver variability remains a well-documented challenge [4,5]. For this reason, ancillary techniques such as immunohistochemistry have become essential in the accurate classification of HM.

The p57 protein, encoded by the CDKN1C gene on chromosome 11p15.5, is a maternally expressed and paternally imprinted cell-cycle inhibitor. Because CHM is purely androgenetic and lacks the maternal genome, p57 expression is absent in villous cytotrophoblasts and stromal cells. In contrast, PHM contains both maternal and paternal genetic contributions and therefore demonstrates positive p57 immunostaining [6]. Recent studies have consistently confirmed the diagnostic utility of p57, reporting sensitivities of 92-98% and specificities exceeding 96% in differentiating CHM from PHM [7,8]. As such, p57 immunostaining has become an indispensable tool that complements histopathology to achieve reliable and reproducible diagnosis.

Given the clinical significance of differentiating complete from partial moles, evaluating the expression pattern of p57 can substantially improve diagnostic accuracy and guide appropriate follow-up and management. This study assesses p57 immunoexpression in HM diagnosed by histopathological criteria and evaluates its diagnostic value in differentiating CHM from PHM.

## Materials and methods

This cross-sectional observational study was conducted in the Department of Pathology, Bangladesh Medical University (BMU), Dhaka, Bangladesh, with additional cases collected from private diagnostic laboratories in Dhaka. The study included females of reproductive age who had been histopathologically diagnosed as CHM, PHM, or placed in an indeterminate category based on routine microscopy. Cases were enrolled through purposive sampling over two years, from September 2019 to August 2021. Formalin-fixed paraffin-embedded tissue blocks with adequate material, along with complete clinical information, were required for inclusion. Cases with inadequate or autolyzed tissue were excluded, as were patients who declined participation. p57 immunohistochemistry was performed in the immunopathology laboratory of BMU, and staining patterns were evaluated in the classified groups.

Sample size calculation

The sample size for the study was estimated using the standard formula for proportions:

n= Z^2^pq/e^2^

where n is the required sample size, p is the estimated prevalence of the outcome of interest, q = (1 - p), Z is the standard normal value corresponding to the 95% confidence level (1.96), and e is the accepted margin of error (0.05). The prevalence of complete mole among molar pregnancies was 34% (i.e., p = 0.34), giving q = 0.66 [9]. Substituting these values yielded an initial sample size of 344.8, which was approximated to 345.

However, due to the limited number of eligible cases available during the study period, primarily influenced by reduced case flow during the COVID-19 pandemic, the modified Cochran formula for small populations was applied:

n_adjusted_= n/1+ (n-1)/N

where N represents the estimated population size (N = 65 cases in the Department of Pathology, BMU, during the study timeframe). Using this correction, the adjusted sample size was calculated as 54.82, which was rounded to a minimum required sample size of 55 cases for the study.

Data collection methods

After getting permission from the Institutional Review Board (IRB) of BMU, known cases of HM received at the Pathology Department, BMU, and private laboratories were collected.

During the collection of specimens, patients’ information and demographic data were recorded systematically in a prepared proforma. Informed written consent was obtained from the patient’s attendant in each case. All the cases were numbered chronologically, and the same number was given to histological as well as immunohistochemical slides.

Case selection and re-evaluation of defined histopathological parameters

Cases diagnosed either as complete and partial HM or HM in the Department of Pathology, BMU, and other private laboratories in Dhaka city were selected for the study by inclusion and exclusion criteria. Paraffin blocks were collected. Sectioning of the paraffin blocks, H&E staining was done according to the standard protocol followed at the Department of Pathology, BMU.

Histopathology lab procedure

Section Cutting

Each block of tissue mounted on the holder was fitted into the microtome machine. A water bath with a regulated temperature of 45 to 50 °C was used for flotation. Sections were cut at 4-5-micron thickness. Ribbons of good sections were selected. The sections were then placed on albumin-coated glass slides. The slides were kept in an inclined position to drain off excess water and allowed to dry at room temperature.

Staining

All the slides for histopathological examination were stained by the routine Hematoxylin and Eosin method in an auto-stainer (Varistain 24-4 Automatic Slide Stainer, Thermo Scientific, Waltham, MA, USA) following standard protocol.

**Figure 1:**
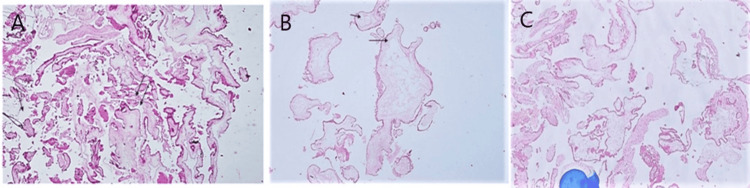
(A) Photomicrograph showing partial hydatidiform mole. Two populations of villi - sclerotic (single arrow) and hydropic (double arrow) - are both seen. (Case no. 50, H&E, 100x). (B) Photomicrograph showing diffuse hydropic degeneration of villi with cistern formation (arrow) in complete hydatidiform mole (Case no. 10, H&E, 100x). (C) Photomicrograph showing indeterminate hydatidiform mole (Case no. 52, H&E, 100x).

Cases were evaluated elaborately, including defined morphological features like trophoblastic hyperplasia, intensity and location, cytologic atypia of trophoblastic cells, pseudoinclusions of trophoblast, cistern formation, scalloping, and fetal vessels in villous stroma. Cases were divided into three groups: CHM, PHM, and the indeterminate group according to the working definition described earlier. Representative sections from each paraffin block were selected for immunohistochemical staining with p57 (Figure 1).

Immunohistochemical Study

Immunohistochemistry was done in the Immunohistochemistry laboratory, BMU. Tissue sections of 3-4 micrometer thickness of formalin-fixed paraffin-embedded tissues were obtained.

Immunohistochemical Analysis of p57

**Figure 2:**
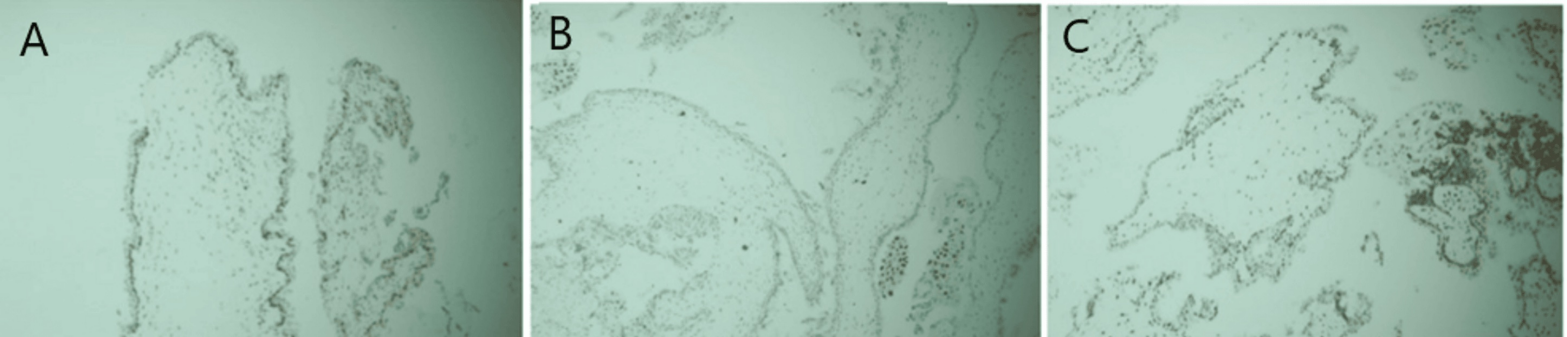
(A) Photomicrograph showing positive nuclear expression of p57 in partial hydatidiform mole (Case no. 43, 200x). (B) Photomicrograph showing negative expression of p57 in cytotrophoblastic and stromal cells of villi in patient of complete hydatidiform mole (Case no. 44, 200x). (C) Photomicrograph showing positive nuclear expression of p57 in histopathologically diagnosed indeterminate cases of hydatidiform mole (Case no. 52, 200x).

Immunohistochemical staining for p57 Kip2 was performed using a rabbit monoclonal primary antibody (clone EP2718(2)) with DAKO REAL™ EnVision™ (HRP Rabbit/Mouse; Agilent Technologies, Santa Clara, CA, USA) as the secondary detection system, and normal placental tissue was used as a positive control. Interpretation of staining was based on nuclear reactivity in villous cytotrophoblastic and stromal cells. Positive expression was defined as distinct nuclear staining in ≥10% of the total cytotrophoblastic cells and villous stromal cells, and such positivity supported a diagnosis of PHM. Cases showing <10% nuclear positivity in these cells were considered negative. Cytoplasmic granular staining in cytotrophoblastic and stromal cells was regarded as negative. Complete absence of nuclear staining in villous cytotrophoblasts and stromal cells confirmed the diagnosis of CHM (Figure 2) [9].

Statistical analysis

The statistical analysis was carried out using SPSS version 22.0 for Windows (IBM Corp., Armonk, NY, USA). Descriptive statistics (frequencies and percentages) were used to summarize the patients’ demographic characteristics and presented in tables, figures, and charts. The frequencies of different entities were expressed as a percentage. The Fisher's exact test was used to analyze the relationship between different categorical variables. Kruskal-Wallis test and Mann-Whitney test both are non-parametric methods. These were done to compare two or more independent datasets. The Mann-Whitney test was used to compare two groups of data, and the Kruskal-Wallis test was used to compare more than two groups of data. P value <0.05 was considered significant. 

Ethical aspect

Ethical clearance for the study was obtained from the Institutional Review Board and the concerned authority, BMU (NO. BSMMU/2020/8537, Date: 27-09-20). Every ethical issue was discussed with the patients regarding the study, and informed written consent was taken from each of them. The entire study subject was thoroughly apprised about the nature, purpose, and implications of the study, as well as the entire spectrum of benefits and risks of the study. All data were secured with confidentiality of the study population. There was no disclosure of the information that may be harmful to the patient.

## Results

A total of 57 histologically diagnosed molar pregnancies were included in the analysis. Of these, 48 cases originated from BMU, and nine cases were obtained from private diagnostic laboratories in Dhaka. All cases were previously classified into CHM, PHM, and indeterminate categories based on defined morphological criteria. Demographic and histopathological characteristics, including age, gestational age at presentation, and specific microscopic features such as villous hydrops, trophoblastic hyperplasia, cytologic atypia, cistern formation, and villous outlines, were documented. p57 immunohistochemistry was successfully performed in all cases, and the pattern of nuclear expression in cytotrophoblasts and villous stromal cells was evaluated. Findings were then correlated with the histopathological grouping to assess diagnostic concordance.

**Figure 3:**
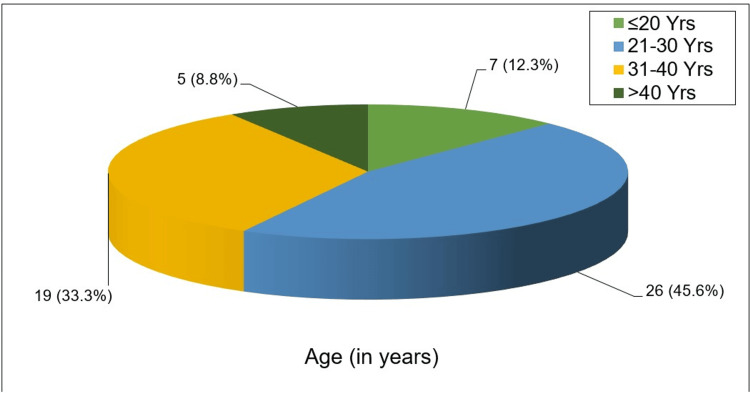
Pie chart showing distribution of the study cases by age (n=57)

In the present study, seven (12.3%) patients were in the 20 or less than 20 age group. Twenty-six (45.6%) patients were in the 21-30 years age group, which represented the highest proportion. Nineteen (33.3%) patients were in the 31-40 years age group, and the lowest age group was >40 years. Only five (8.8%) patients were in this group. The mean age of the total 57 study participants was 29.0 years (SD:7.7) (Figure 3).

Distribution of study cases by gestational age showed that the proportion of study cases was highest among the gestational age of 12 to 16 weeks, which was 57.89% (33 cases). The other two groups were <12 weeks and 17-20 weeks, and the proportion of study cases was same for both groups, which was 21.05% (12 cases in each group). The mean gestational age was 13.96±3.43 weeks (Table 1). 

**Table 1:** Distribution of the study cases by gestational age (n=57)

Gestational Age (weeks)	Number of Cases (n)	Percentage (%)
< 12 weeks	12	21.05%
12–16 weeks	33	57.89%
17–20 weeks	12	21.05%
Total	57	100%

Bad obstetric history was one of the demographic variables. It was observed that almost one-third (33.3%) of patients had a bad obstetric history. Though the majority of patients did not have a bad obstetric history, the proportion was higher (66.7%, 38 cases) (Table 2).

**Table 2:** Distribution of study cases by bad obstetric history (n=57)

Bad obstetric history	Number of patients (n)	Percentage (%)
Yes	19	33.3
No	38	66.7
Total	57	100.0

The association between P57 expression and bad obstetric history was observed. Only 5.3% (1/19) of patients with p57 expression positive had a bad obstetric history, whereas 94.7% (18/19) of patients with p57 expression negative had a bad obstetric history. There was a significant (p<0.05) association between p57 expression and bad obstetric history (Table 3).

**Table 3:** Association of p57 expression with bad obstetric history (n=57) s = significant, CHM = complete hydatidiform mole, PHM = partial hydatidiform mole p-value reached from Fisher's exact test

Bad obstetric history	p57 expression				p value
	Positive (PHM) (n=15)		Negative (CHM) (n=42)		
	n	%	n	%	
Yes (n=19)	1	5.3	18	94.7	0.011^s^
No (n=38)	14	36.8	24	63.2	

Different histopathological parameters were used to subclassify the HM as shown in Table 4. It was observed that all (36, 100%) patients had diffuse hydropic degeneration of villi in CHM, one (7.7%) in PHM, and six (75%) in the indeterminate group. One of the histologic parameters was trophoblastic hyperplasia, intensity, and location, where all CHM had marked trophoblastic hyperplasia. Twenty-six cases (72.2%) of CHM had marked, circumferential hyperplasia, and 10 (27.8%) CHM had marked, polar hyperplasia. In PHM the maximum proportion of cases (10, 76.9%) had mild, polar trophoblastic hyperplasia, and 50% of the indeterminate group had mild, circumferential trophoblastic hyperplasia. The majority (86.1%) of patients of CHM had marked cytologic atypia of trophoblastic cells, which was not found in PHM, and there was one (12.5%) in the indeterminate group. Pseudoinclusions of trophoblast were not found in any cases of CHM, five (38.5%) cases in PHM, and three (37.5%) cases in the indeterminate group. The majority (34, 94.4%) of cases had cistern formation in CHM, one (7.7%) in PHM, and four (50%) in the indeterminate group. One (2.8%) case had scalloping in CHM, 13 (100%) in PHM, and seven (87.5%) in the indeterminate group. Nearly one-third (30.8%) of cases had fetal vessels in villous stroma in PHM, whereas fetal vessels in villous stroma were not found in CHM and indeterminate cases. The differences in histopathological findings were statistically significant (p<0.05) among the three groups.

**Table 4:** Association between histopathological diagnosis and histopathological findings (n=57) s = significant, CHM = complete hydatidiform mole, PHM = partial hydatidiform mole, Cir = circumferential p-value reached from Fisher's exact test

Histopathological findings	Histopathological diagnosis						p value
	CHM (n=36)		PHM (n=13)		Indeterminate (n=8)		
	n	%	n	%	n	%	
Hydropic degeneration of villi							
Focal	0	0.0	12	92.3	2	25.0	0.001^s^
Diffuse	36	100.0	1	7.7	6	75.0	
Trophoblastic hyperplasia, intensity and location							
Marked, Cir	26	72.2	0	0	0	0.0	
Marked, Polar	10	27.8	0	0	2	25.0	0.001^s^
Mild, Cir	0	0.0	3	23.1	4	50.0	
Mild, Polar	0	0.0	10	76.9	2	25.0	
Cytologic atypia of trophoblastic							
Mild	5	13.9	13	100.0	7	87.5	0.001^s^
Marked	31	86.1	0	0.0	1	12.5	
Pseudoinclusions of trophoblast							
Present	0	0.0	5	38.5	3	37.5	0.001^s^
Absent	36	100.0	8	61.5	5	62.5	
Cistern formation							
Present	34	94.7	1	7.7	4	50.0	0.001^s^
Absent	2	5.6	12	92.3	4	50.0	
Scalloping							
Present	1	2.8	13	100.0	7	87.5	0.001^s^
Absent	35	97.2	0	0.0	1	12.5	
Fetal vessels in villous stroma							
Present	0	0.0	4	30.8	0	0.0	0.001^s^
Absent	36	100.0	9	69.2	8	100	

Out of 36 histopathologically diagnosed cases of CHM, four (11.1%) cases exhibited positive p57 nuclear expression, and 32 (88.9%) showed negative p57 nuclear expression. Among 13 PHM (histopathologically diagnosed), nine (69.2%) showed immunopositivity for p57, and the remaining four (30.8%) exhibited p57 immunonegativity. Histopathologically diagnosed indeterminate group had a total of eight cases. Among those six (75%) had negative immunoexpression for p57, and two (25%) had positive immunoexpression for p57. The difference was statistically significant (p<0.05) (Table 5).

**Table 5:** Association of histopathological diagnosis with p57 expression (n=57) CHM = complete hydatidiform mole, PHM = partial hydatidiform mole

Histopathological Diagnosis	p57 Positive (PHM)		p57 Negative (CHM)		p value
	n	%	n	%	
Partial Hydatidiform Mole (n=13)	9	69.2	4	30.8	
Complete Hydatidiform Mole (n=36)	4	11.1	32	88.9	0.001ᵗ
Indeterminate (n=8)	2	25.0	6	75.0	
Total (n=57)	15	26.3	42	73.7	

## Discussion

The present study evaluated 57 histologically diagnosed molar pregnancies obtained from both a tertiary academic center and private diagnostic laboratories. Standard morphological criteria were used to classify cases into CHM, PHM, and indeterminate types. Detailed clinical, biochemical, and histopathological features were documented, and all cases underwent p57 immunohistochemical analysis. This comprehensive approach mirrors modern diagnostic protocols, integrating ancillary markers with morphology to achieve greater diagnostic accuracy in gestational trophoblastic disease [10,11].

The age distribution showed that nearly half of the patients (45.6%) were between 21 and 30 years, with a mean age of 29.0 years. This is consistent with global and regional data showing that the majority of molar pregnancies occur in women of reproductive age, particularly between 20 and 35 years [12]. Although very young and advanced maternal age (>40 years) are recognized risk factors, the lowest proportion in the current study belonged to the >40 age group, similar to the findings of the recent Asian population, where delayed detection and demographic changes influence age patterns [13]. Comparable studies from South Asia also report mean ages ranging from 26 to 30 years, reflecting similar reproductive profiles [14].

More than half of all cases (57.9%) presented between 12 and 16 weeks of gestation. This distribution aligns with the common clinical trend that molar pregnancies are often diagnosed in the late first or early second trimester when symptoms such as abnormal bleeding or abnormal ultrasound findings become more prominent [15]. The equal proportions in the <12-week and 17-20-week groups suggest a mixed pattern of early antenatal care access among patients. Recent retrospective studies from various regions likewise report peak detection of molar gestations around 11-15 weeks due to increased utilization of routine sonography [16].

In this study, one-third (33.3%) of the patients had a history of adverse obstetric outcomes. Previous pregnancies complicated by miscarriage, stillbirth, or prior molar pregnancy are known contributors to increased risk of subsequent HM [17]. Although the majority of patients (66.7%) had no such history, the proportion with previous pregnancy loss is comparable to findings reported in several recent studies from Southeast Asia and the Middle East [18]. These observations underline the importance of careful follow-up in women with previous reproductive complications.

A significant association (p=0.011) was found between p57 expression and bad obstetric history. Most patients with a positive history (94.7%) belonged to the p57-negative group (CHM), whereas only one out of 19 belonged to the p57-positive group (PHM). This indicates that CHM may be more common among women with prior adverse reproductive outcomes. Previous studies have similarly observed that recurrent reproductive loss is more frequently associated with CHM compared to PHM, possibly due to underlying genetic susceptibility to androgenetic conceptions [19]. The current findings reinforce the value of incorporating p57 immunostaining in evaluating recurrent molar gestations.

The classical morphological differences between CHM, PHM, and indeterminate cases were clearly reflected in this study. CHM showed diffuse villous edema (100%), marked trophoblastic hyperplasia, and prominent cytologic atypia, findings strongly supported by contemporary guidelines such as the WHO 2020 classification [20]. PHM, on the other hand, demonstrated focal hydropic changes, mild trophoblastic proliferation, scalloping, and occasional fetal vessels hallmarks described in recent pathology literature [21]. The indeterminate category displayed overlapping features, a diagnostic challenge consistently reported in recent research where morphology alone can misclassify up to 10-15% of cases [22]. The significant p-values across all parameters underscore the reliability of these morphological criteria while highlighting the necessity of ancillary tools such as p57 immunohistochemistry.

The study revealed CHM as the most common subtype (63.2%), followed by PHM (22.8%) and indeterminate cases (14%). This pattern closely follows epidemiologic trends reported from other South Asian and global populations, where CHM accounts for approximately 60-75% of molar gestations [9]. The proportion of indeterminate cases in the present study also aligns with recent evidence showing that a notable minority of cases exhibit overlapping features when assessed solely by morphology [23]. This reinforces the importance of integrating immunohistochemistry for improved diagnostic classification.

In the current study, 73.7% of cases showed negative p57 expression (CHM) while 26.3% were positive (PHM). These proportions closely mirror findings from various international studies confirming that CHM is typically p57-negative due to its androgenetic origin, whereas PHM, containing a maternal genome, exhibits nuclear positivity [24]. This distribution further supports the practical utility of p57 IHC as a highly reliable marker in distinguishing CHM from PHM.

A significant association (p=0.001) was found between histopathological diagnosis and p57 expression. While 88.9% of CHM cases showed the expected p57-negative pattern, a small proportion (11.1%) demonstrated unexpected positivity likely attributable to misclassification or early gestational specimens with ambiguous features. Similarly, 69.2% of PHM cases showed p57 positivity, in agreement with genetic expectations. The indeterminate group also displayed mixed findings, reflecting the documented limitations of morphology alone [25]. Recent studies emphasize that up to 10% of cases may show discordance between morphology and p57 due to rare genetic variants, early gestational sampling limitations, or fixation issues [26]. In this context, the current findings underscore the strong, but not absolute, diagnostic value of p57 immunohistochemistry.

A key limitation of this study is that the study population was recruited exclusively from Dhaka city, which may limit the generalizability of the findings to the broader population of Bangladesh, particularly to rural and peripheral healthcare settings where diagnostic facilities and case profiles may differ. Additionally, the relatively small sample size may have reduced the statistical power of the analysis and limited the ability to detect subtle variations in p57 immunoexpression between complete and partial hydatidiform moles. Other potential limitations include the single-center study design and the inherent constraints of a cross-sectional approach, which precluded assessment of longitudinal outcomes such as progression to gestational trophoblastic neoplasia. Despite these limitations, the study provides valuable insight into the diagnostic utility of p57 immunostaining in differentiating complete from partial hydatidiform moles in a resource-limited setting.

## Conclusions

This study demonstrates that p57 immunohistochemistry is a valuable diagnostic tool for accurately distinguishing CHM from PHM and resolving cases with indeterminate morphology. The photomicrographic evidence showed consistent positive p57 nuclear staining in villous cytotrophoblasts and stromal cells of partial moles, while complete moles exhibited characteristic absence of staining. Indeterminate cases, which frequently pose diagnostic difficulties using morphology alone, were also successfully classified following p57 evaluation. The strong correlation between histopathological features and p57 expression confirms the utility of this biomarker in routine practice. Incorporating p57 immunostaining alongside conventional histopathology enhances diagnostic precision, guides appropriate clinical management, and reduces the risk of misclassification. Further integration of molecular genotyping may strengthen the accuracy of diagnosing atypical or discordant cases.
